# LncRNA ENST869 Targeting Nestin Transcriptional Region to Affect the Pharmacological Effects of Chidamide in Breast Cancer Cells

**DOI:** 10.3389/fonc.2022.874343

**Published:** 2022-04-04

**Authors:** Xiuyan Feng, Han Han, Yarui Guo, Xue Feng, Shanchun Guo, Weiqiang Zhou

**Affiliations:** ^1^ Medical Administration Division, The Second Affiliated Hospital of Shenyang Medical College, Shenyang City, China; ^2^ Department of Biochemistry and Molecular Biology, Shenyang Medical College, Shenyang City, China; ^3^ Department of Pathogen Biology, Shenyang Medical College, Shenyang City, China; ^4^ RCMI Cancer Research Center, Xavier University of Louisiana, New Orleans, LA, United States

**Keywords:** biomarker, LncRNA, Chidamide, Nestin, anti-cancer drug resistance

## Abstract

Breast cancer is one of the leading threats to the health of women. It has the highest incidence and mortality in women worldwide. Although progress has been made in the development and application of anti-breast cancer drugs such as Chidamide and others, the occurrence of drug resistance limits the effective application of chemotherapies. The purpose of this study is to explore the role of LncRNA in the pharmacological effect of Chidamide in breast cancer therapy. The human breast cancer MCF-7 or MDA-MB-231 cells were used as the research cell models. The RNA library screening and high-throughput sequencing comparative analysis was conducted. The binding of LncRNA and its downstream target genes in RNA and protein levels was tested. The results showed that the expression of LncRNA ENST869 in cells treated with Chidamide increased significantly, as demonstrated by real-time PCR and cell viability assay. RNAplex analysis showed that LncRNA ENST869 and Nestin mRNA may interact. RNA interference and Western blot analysis indicated that LncRNA ENST869 could target and regulate the expression of Nestin. Luciferase assay and RNA-protein pulldown showed that LncRNA ENST869 affected Nestin transcription. There might be a highly active binding region of LncRNA ENST869 in regulating Nestin transcriptional activity within the site of 250 bp upstream of the transcription starting point of Nestin. In addition, LncRNA ENST869 did not directly interact with Nestin protein to affect its activity. In conclusion, our results demonstrated that LncRNA ENST869 could affect the function of Nestin in breast cancer cells treated with Chidamide. Nestin is a key player in influencing the pharmacological activity of Chidamide and an essential factor in drug resistance of breast cancer cells.

## Introduction

The incidence of breast cancer ranks the highest among all cancers in women worldwide (1, 2). With the intensive study on the structure and function of the human genome, it was found that histone acetylation regulation is an important epigenetic modification, which regulates the dynamic balance between histone acetyltransferase (HAT) and histone deacetylase (HDAC) and plays an important role in the occurrence and development of breast cancer (3–5). The gene transcription activity instead of DNA sequence mutation and stable genetic expression regulation has gradually become an intriguing topic of current research (6).

Chidamide is a third-generation histone deacetylation inhibitor (HDACi) (7). It has better efficacy than the second-generation HDACi anti-cancer drugs such as TSA and SAHA (8). However, its clinical application has inherent defects similar to other common anti-tumor drugs such as drug resistance (9).

Long non-coding RNA (LncRNA) is a kind of RNA molecule that does not encode protein and has a length of 200–10,000 nucleotides (10–12). At present, there are thousands of LncRNA genes involved in mammalian gene activities. The mechanism of LncRNA in regulating gene expression is mainly achieved *via* epigenetics, transcriptional regulation, and post-transcriptional regulation (13, 14). In particular, the transcription regulation of LncRNA can interfere with the expression of its downstream genes and impact the activity of other transcription factors. Moreover, some LncRNAs can form double-stranded RNA complexes with targeted mRNAs to mask the cis-acting elements of mRNA and regulate its gene expression (15).

In this study, Chidamide is applied to estrogen receptor (ER) positive MCF-7 and triple-negative MDA-MB-231 breast cancer cells in order to explore the role of LncRNA in the regulatory mechanism in breast cancer growth inhibition. The goal is to provide a theoretical basis and experimental data for further clarifying the molecular mechanism of action of LncRNA and distinctive factors.

## Materials and Methods

### Cell Lines and Reagents

Human MCF-7 or MDA-MB-231 cells were obtained from American Type Culture Collection (ATCC) (Manassas, VA). Leibovitz’s L-15 medium, RPMI-1640 medium, Fetal Bovine Serum (FBS) and, Penicillin–streptomycin Cocktails were purchased from Life Technologies (Austin, TX). Chidamide was supplied by Sigma-Aldrich (St. Louis, MO). Luciferase Assay System and CellTiter 96^®^ AQueous One Solution Cell Proliferation assay were from Promega (Madison, MI). Muse Count & Viability Kit was from Millipore (Darmstadt, Germany). High Pure RNA Isolation Kit and Transcriptor First Strand cDNA Synthesis Kit were given from Roche Diagnostics GmbH (Mannheim, Germany). 5’RACE System for Rapid Amplification of cDNA Ends, LightShift Chemiluminescent RNA EMSA Kit, Power SYBR Green PCR Master Mix, RIPA Cell Lysis Buffer and BCA Protein Assay Kit were from Life Technologies (Austin, TX). Polyclonal anti-Nestin antibody and polyclonal anti-β actin antibody were obtained from Abcam Inc. (Cambridge, MA). LncRNA ENST869 siRNA candidates were designed and synthesized by RiboBio (Guangzhou, China). Nestin siRNA(h) was purchased from Santa Cruz Biotechnology (Santa Cruz, CA). Other chemicals were from Sangon Biotech (Shanghai, China).

### Cell Culture

MCF-7 or MDA-MB-231 cells were grown in Leibovitz’s L-15 medium or RPMI-1640 medium, respectively, with supplementation of 15% fetal bovine serum (FBS), 100 U/ml penicillin and 100 μg/ml streptomycin. All the cells were maintained at 37°C with 5% CO_2_ and 95% humidity. The cells were seeded at a density of 1.0 × 10^4^ cells/ml in a 96-well plate, 5.0 × 10^5^ cells/ml in a 6-well plate and 1.5 × 10^7^ cells/ml in a 100 mm dish. The cells were grown to 70–80% confluence and starved for 24 h in basal medium (with DMSO) without FBS and treated with different compounds.

### MTT and Cell Viability Assay

MCF-7 or MDA-MB-231 cells were plated in a 96-well plate (5.0 × 10^3^ cells/ml). After 24 h of starvation, the cells were treated with different concentrations of Chidamide (0, 20, and 100 μM) for 48 h incubation. The same concentrations of DMSO were added as a control. The CellTiter 96^®^ AQueous One Solution Cell Proliferation Assay (MTT) was used, and absorbance was measured at 490 nm on a microplate reader.

Muse Count & Viability Reagent was used to assess cell viability. Approximately 2 × 10^5^ of harvested cells (50 μl cell suspension) was added with 450 μl Count & Viability reagent. The results were obtained using the Muse Count & Viability software module in Muse Cell Analyzer, and the statistics showed the concentrations and percentages of viable and dead cells.

### Breast Cancer Tumor Xenograft Models

The female nude mice were subcutaneously injected with 1 × 10^7^ MDA-MB-231 cells suspended in 100 μl PBS. Length (L) and width (W) of the tumor were determined by a vernier caliper. The tumor volume (V) was calculated according to the equation: V = L × W^2^/2.

Once the tumor reached 50 mm^3^, tumor-bearing mice were randomized into treatment groups. The mice were intravenously administrated with Chidamide (12 mg/kg Chidamide) and saline as a negative control for every two days (total of 12 injections). The tumor size and body weight of the mice were monitored every 2 days. On day 23, the mice were sacrificed. The tumors were harvested and weighted.

### siRNA Transfection

siRNAs were transfected into MDA-MB-231 or MCF-7 cells maintained in 6-well plates using Lipofectamine 3000 transfection reagent. Approximately 6.6 μl siRNA was mixed with 125 μl medium without serum for 5 min, and the 3.75 μl Lipofectamine 3000 reagent was mixed with 125 μl medium without serum for 5 min. These two reagents were mixed and incubated at room temperature for 5 min. The cell culture medium was removed from each well of the 6-well plates, and the mixture of siRNA and Lipofectamine 3000 was added to each well. Approximately 1 ml medium without serum was added with cells for 12 h of incubation. Finally, the transfection mixture was removed and the cells were cultured with 2 ml of medium with serum for 24 h.

### Luciferase Assay

MDA-MB-231 cells were treated with Chidamide or siRNAs as described before. Growth medium was carefully removed, and the cells were rinsed with PBS. Approximately 300 μl 1× lysis buffer was added to cover the cells. After rocking the dishes several times, attached cells were scraped from the dish. Cells were then transferred to a 1.5 ml tube and kept in ice for 1 min. The luminometer was programed to perform a 2-second measurement delay followed by a 10-second measurement read for luciferase activity. Approximately 20 μl cell lysate was then mixed with 100 μl luciferase assay reagent. The samples were analyzed on Glomax^®^ 96 luminometer and initiated reading.

### cDNA Synthesis and Real-Time PCR Analysis

Total RNA was extracted from MCF-7 or MDA-MB-231 cells using High Pure RNA Isolation Kit according to the instructions of the manufacturer. RNA quantitation was performed *via* real-time PCR. The total RNA was reverse-transcribed with Transcriptor First Strand cDNA Synthesis Kit and amplified by Power SYBR Green PCR Master Mix in an Applied Biosystems 7500 real-time PCR system. The primers were designed by Primer 3 suit and libraries. The sequences of primers are shown in **Table 1**. Data normalization was based on correcting all *C*_t_ values for the average *C*_t_ values of GAPDH gene present in the reaction. Three independent biological replicates were performed.

**Table 1:** The primers used are summarized.

Gene Name	Primer Orientation	Sequence
LncRNA Primer1	Forward	5’-TCAGGGACAGGGCAGTATTC-3’
	Reverse	5’-GGGCTCCATCATCTTCTCTG-3’
LncRNA Primer2	Forward	5’-GTAGGCCTCGTTCACCTTGA-3’
	Reverse	5’-GGGTCAAGTGGACTTTCCTG-3’
LncRNA Primer3	Forward	5’-GAGCCGGCTGGAACTTAAC-3’
	Reverse	5’-CCGGAAGGAGGGATCCTG-3’

Nestin Forward primer: 5’-GCTGAAGCCCTGGGGAAAGT-3’.

Reverse primer: 5’-CCAGGGGAGTGGAGTCTGGA-3’.

GAPDH Forward primer: 5’-ACAGTCAGCCGCATCTTCTT-3’.

Reverse primer: 5’-ACGACCAAATCCGTTGACTC-3’.

### Western Blot Analysis

The MCF-7 or MDA-MB-231 cell pellets collected from 6-well plates were incubated in RIPA buffer containing 0.1 mg/ml protease inhibitor. The cellular lysate was rotated for 2 h at 4°C followed by centrifugation for 10 min at 14,000*g* at 4°C. Proteins were quantified using BCA protein assay kit. For immunoblotting, 20 μg proteins were separated by SDS-polyacrylamide gels electrophoresis and transferred to PVDF membranes. Western blot analyses were performed using the antibodies described above. The level of β-actin was used as loading controls. Protein bands were detected using ECL western blot substrate and exposed on DNR MF-Chemi Bio-Imaging Systems.

### LncRNA Sequencing and Analyzing

Total RNA was extracted and ribosomal RNA was removed using the Ribo-Zero Kit (Epicentre, Madison, WI, USA). Fragmented RNAs (the average length was approximately 200 bp) were subjected to the first strand and second strand cDNA synthesis followed by adaptor ligation and enrichment with a low-cycle according to instructions of NEBNext^®^ Ultra RNA Library Prep Kit for Illumina (NEB, USA). The purified library products were evaluated using the Agilent 2200 TapeStation and Qubit^®^2.0 (Life Technologies, USA). The libraries were paired-end sequenced (PE150, Sequencing reads were 150 bp) at RiboBio (Guangzhou, China) using the IlluminaHiSeq 3000 platform.

For identification of new LncRNA, the raw data were first filtered to remove low-quality reads. Then, the clean data that passed repeated testing was assembled using the StringTie based on the reads mapped to the reference genome. The assembled transcripts were annotated using the GffCompare program. The unknown transcripts were used to screen for putative LncRNAs. Transcripts with lengths above 200 nucleotides with predicted ORF shorter than 300 nucleotides were selected as LncRNA candidates.

For differential expression analysis, the statistically significant DE genes were obtained by an adjusted P-value threshold of 1 using the DEGseq software. Finally, a hierarchical clustering analysis was performed using the R language package gplots according to the TPM values of differential genes in different groups. Different colors represent different cluster of information, such as the similar expression pattern in the same group, which includes similar functions or participating in the same biological process.

All differentially expressed mRNAs were selected for GO pathway analyses. GO was performed with KOBAS3.0 software. GO provides label classification of gene function and gene product attributes (http://www.geneontology.org). GO analysis covers cellular component (CC) domains.

### Northern Blot Assay

The synthesis of the RNA probe used for northern blot was performed using the 5’ RACE Kit following the protocol. After first-strand cDNA synthesis from total or poly(A)^+^ RNA using a gene-specific primer (GSP1), a homopolymeric tail was added to the 3’-end of the cDNA using TdT and dCTP. PCR amplification was accomplished using Taq DNA polymerase, a nested, gene-specific primer (GSP2) that anneals to a site located within the cDNA molecule, and a novel deoxyinosine-containing anchor primer (Abridged anchor primer) provided with the system. The sequences of GSP1 single primers: 5’-GACTCTGAGAGCAGGGCAAG-3’. The sequences of GSP2 single primers: 5’-CCGACTTAACTGACCCTCTG’.

Northern blot was performed according to the protocol described previously. In brief, a 15 μg aliquot of total RNA was resuspended in 5 μl of RNase-free H_2_O, supplemented with two volumes of RNA loading buffer. RNA samples were resolved on a 0.8% formaldehyde-agarose gel, capillary transferred onto positively charged nylon transfer membrane, rinsed with H_2_O and UV crosslinked at 1,200 mJ (0.1 mJ/cm^2^) with 60 s. North2South^®^ Chemiluminescent Hybridization and Detection Kit was used and the membrane was hybridized overnight to the Bio-labeled RNA probe at a final concentration of 150 ng/ml. Chemiluminescence detection was performed according to the instructions of the manufacturer.

### EMSA Assay

To demonstrate a functional EMSA, the LightShift Chemiluminescent RNA EMSA Kit was used. Approximately 20 μl binding reaction contained biotin-labeled target RNA, unlabeled target RNA and protein extract. Reactions were electrophoresed, transferred, and crosslinked of binding reactions to nylon membrane according to the steps in the protocol. The biotin end-labeled RNA is detected using the Streptavidin Horseradish Peroxidase Conjugate and a highly sensitive chemiluminescent substrate.

### Data Analysis

All data in the text and figures are provided as means ± S.D. The results were analyzed by a one-way analysis of variance (ANOVA), followed by Tukey *post hoc* comparisons. All analyses were performed using the Statistical Package for Social Science (SPSS) software v22.0 (IBM, Armonk, NY). *P <*0.05 was considered significant.

## Results

### Chidamide Constrains Breast Cancer Cell Growth

**Figure 1:**
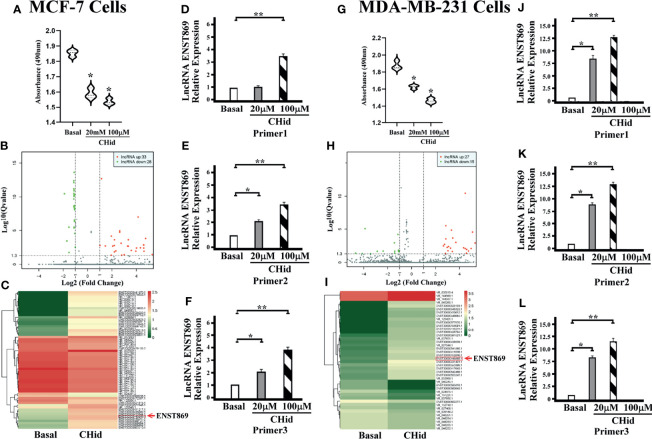
Chidamide inhibits breast cancer cell growth. MCF-7 or MDA-MB-231 cells were cultured for 48 h in the presence of 20 or 100 μM Chidamide. The same concentrations of DMSO were used as control. The cell viability was measured by MTT assay **(A**, **G)**. The LncRNA fragments of breast cancer cells were screened by high-throughput RNA sequencing (RNAseq), and hierarchical clustering analysis was performed using the R language package gplots according to the TPM values of differential genes in different groups. The abscissa represents the fold changes of gene expression in different samples, and the ordinate represents the statistical significance of the difference in gene expression. Volcanic diagram showing the differential gene expression between samples **(B**, **H)**. Heat map of differential gene expression analysis between samples **(C**, **I)**. We designed three pairs of primers (Primers 1–3) to verify the real levels of LncRNA ENST869 in the treated cells [**(D**–**F)** for MCF-7, **(J**–**L)** for MDA-MB-231]. Data was presented as means ± S.D. for three independent experiments and analyzed using SPSS software, *20 μM Chidamide versus Basal at *p <* 0.05, **100 μM Chidamide versus Basal at *p <* 0.05.

First, we measured the pharmacological effects of Chidamide on breast cancer cell growth. According to the previous experimental results (16), MCF-7 and MDA-MB-231 cells were treated with 20 μM Chidamide for 48 h. The same concentration of DMSO was used as the positive control. Cell viability assay showed that Chidamide inhibited cell activity (**Figures 1A, G**). To further understand the mechanism of action of Chidamide, after screening positive LncRNA by high-throughput RNA sequencing (RNA-seq) in MCF-7 and MDA-MB-231 cells, we analyzed the known mRNA from the sequencing results with DEGseq (an R package used to screen the differential gene expression in different samples). From the volcano diagram of mRNA differential expression (**Figures 1B, H**), it was found that the expressions of 33 and 27 LncRNA molecules were increased in MCF-7 and MDA-MB-231 cells with Chidamide treatment respectively, accompanied by a decrease in the expressions of 28 and 15 LncRNA molecules in both cell lines, respectively. Heatmap analysis showed that an LncRNA molecule named ENST00000448869.1 (referred to as ENST869 thereafter) was consequentially produced in both cell lines (**Figures 1C, I**). Finally, we obtained the full sequence of LncRNA ENST869 in the Ensembl genome browser and designed three pairs of primers (Primer 1-3) based on different regions of the LncRNA sequence by Primer3 software to verify the software deduction. The primer sequences are listed in **Table 1**. From the results of real-time quantitative PCR, we asserted that ENST869 is over-expressed in MCF-7 (**Figures 1D–F**) and MDA-MB-231 cells (**Figures 1J–L**).

### LncRNA ENST869 Promotes the Expression of Nestin mRNA

**Figure 2:**
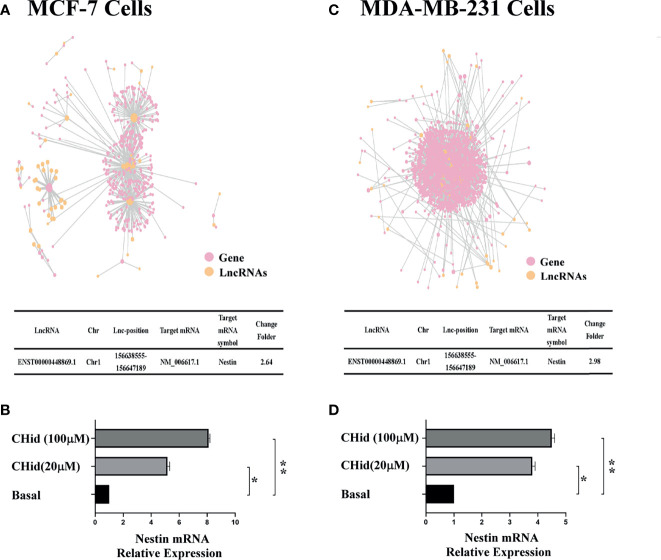
LncRNA ENST869 promotes the expression of Nestin mRNA. The potential crosstalk between LncRNA and mRNAs was predicted through RNAplex software. Real-time PCR was used to verify the level of Nestin mRNA in Chidamide treated cells. Data was presented as means ± S.D. for three independent experiments and analyzed using SPSS software, *20 μM Chidamide versus Basal at *p <* 0.05, **100 μM Chidamide versus Basal at *p <* 0.05 [**(A**, **B)** for MCF-7, **(C**, **D)** for MDA-MB-231].

The action network of LncRNAs was established using the RNAplex software, to predict the relationship between LncRNAs and their targeted mRNAs. The results were shown in **Figures 2A, C**. We integrated the data of differential LncRNAs and its adjacent mRNAs (<10 KB) and identified a potential target, Nestin. Real-time PCR indicated that Nestin mRNA was hugely accumulated in Chidamide treated cells (**Figures 2B, D**).

In order to clarify the operation pattern of LncRNA ENST869, we designed three siRNA fragments followed in the whole sequence of LncRNA ENST869 to silence this LncRNA. The sequences of these siRNAs are as follow:

siRNA1:5’-GCTCTCCTGATCAATACAT-3’; siRNA2:5’-CCAAAGTCTCCCAGTCAAT-3’; siRNA3:5’-GGCTGGAACTTAACGCTGT-3’.

**Figure 3:**
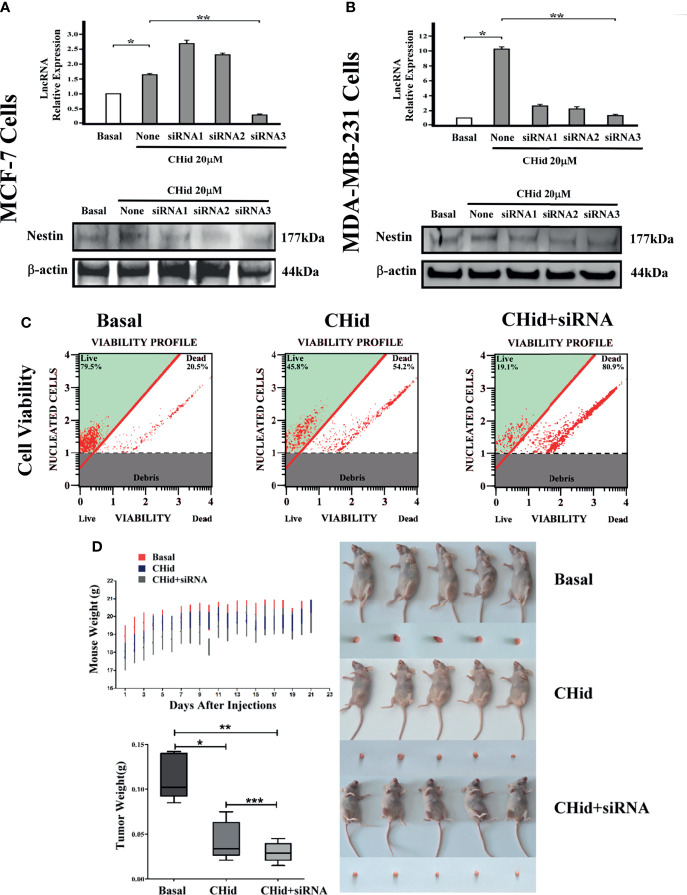
Silencing LncRNA ENST869 increases the pharmacological effect of Chidamide. Three siRNA fragments designed for LncRNA ENST869 were screened by Real-time PCR and Western blot was done to detect the Nestin protein levels in **(A)** MCF-7 cells and **(B)** MDA-MB-231 cells. In addition, cell viability **(C)** assays and tumor xenograft models **(D)** for MDA-MB-231 cells verified the knockdown effects of LncRNA ENST869. Data was presented as means ± S.D. for three independent experiments and analyzed using SPSS software, *20 μM Chidamide versus Basal at *p <*0.05, **20 μM Chidamide with LncRNA siRNA versus Basal at *p <*0.05, ***20 μM Chidamide versus 20 μM Chidamide with LncRNA siRNA.

**Figures 3A, B** showed that siRNA3 presented an ideal inhibition on LncRNA ENST869 in both cell lines. Interestingly, Western blot demonstrated that Nestin protein was also obviously interfered along with the functional loss of LncRNA ENST869. This result indicates that there is a correlation between LncRNA ENST869 and Nestin. Cell viability and nude mice bearing tumor experiment results showed that, when LncRNA ENST869 was silenced, the number of living cells was drastically reduced, and the tumorigenicity in mice was declined, implying that the anti-cancer efficacy of Chidamide was enhanced (**Figures 3C, D**).

### Silencing Nestin mRNA Does Not Affect LncRNA ENST869

**Figure 4:**
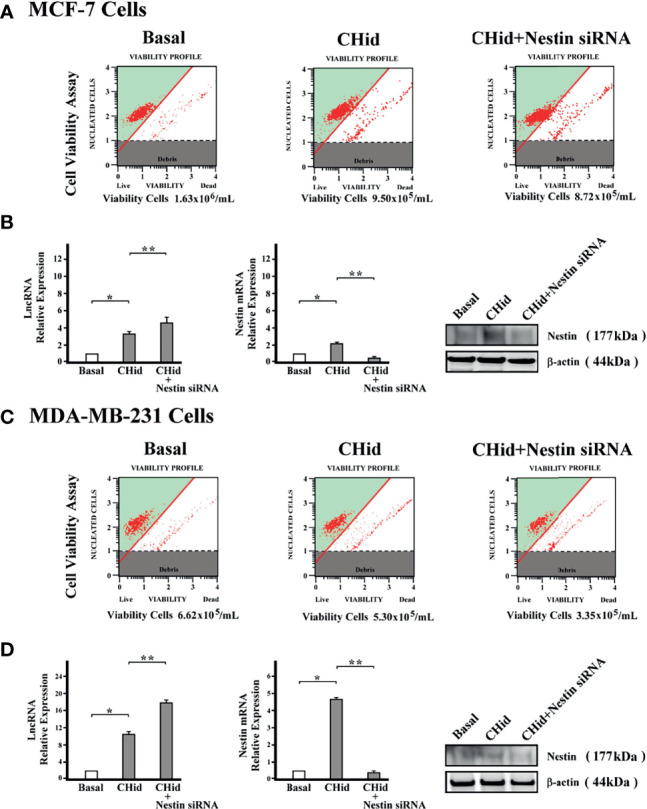
Knockout of Nestin mRNA does not repress the expression of LncRNA ENST869. After transfecting the special Nestin siRNA and Chidamide treatment in breast cancer cells, cell viability was assessed **(A, C)**. Real-time PCR and Western blot detected the mRNA and protein level of LncRNA ENST869 and Nestin **(B, D)**. Data was presented as means±S.D. for three independent experiments and analyzed using SPSS software, * 20 mM Chidamide versus Basal at p<0.05, ** 20 mM Chidamide with Nestin siRNA versus Chidamide at *p <*0.05.

To discern whether Nestin has a feedback regulation on LncRNA ENST869, we used specific siRNA to silence Nestin mRNA. **Figure 4** displayed that breast cancer cell activity was suppressive; however, LncRNA ENST869 was not affected.

### LncRNA ENST869 Binds to the Specific Region of Nestin Transcription Promoter to Regulate Its Transcriptional Activity

**Figure 5:**
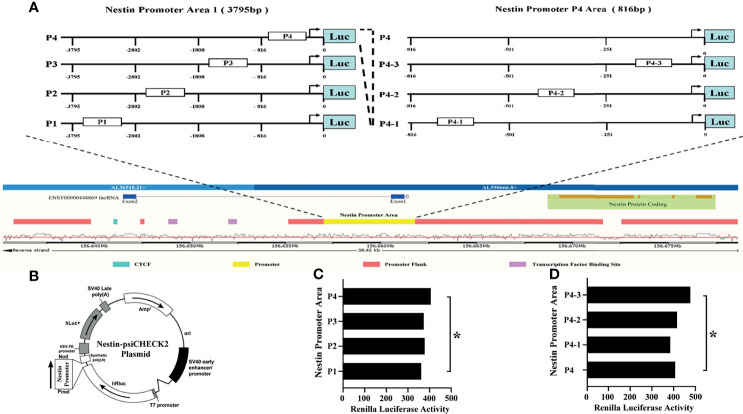
LncRNA ENST869 binds to the specific region of the Nestin promoter to regulate its transcription activity. The Ensembl database was used to search the genomic arrangement of LncRNA ENST869 and Nestin promoter **(A)**. The sequences of Nestin promoter domains were subcloned into psiCHECK plasmid, and the activity of Luciferase was detected in cells co-transfected with LncRNA ENST869 **(B**–**D)**. Data was presented as mean ± S.D. for three independent experiments. **(C)** * P4 versus P1 at p<0.05, **(D)** * P4-3 versus P4 at p<0.05.

Next, to explore the binding between ENST869 and Nestin mRNA, we examined the genome arrangement of LncRNA ENST869 and Nestin in the Ensembl database. As shown in **Figure 5A**, LncRNA ENST869 works in the pattern of antisense RNA. It has two exons, of which exon1 is adjacent to Nestin promoter regions, indicating that both of them have the binding possibility at the genomic level. Therefore, we designed the Nestin promoter into four regions (Named P1–P4), and synthesized those DNA sequences respectively. The primers used are listed in **Table 2**. After being subcloned into psiCHECK plasmid as shown in **Figure 5B** (Suppl. for plasmid construction), Luciferase activity was detected in cells with LncRNA ENST869 co-transfection. The results showed that the Nestin P4 promoter region showed high luciferase activity (**Figure 5C**). Furthermore, we analyzed the P4 promoter region into p4-1, p4-2 and p4-3 in detail. **Figure 5D** hinted that p4-3 possessed the highest luciferase activity. It indicates that within the 250 bp upstream of the starting point of Nestin transcription, there is a primary region of LncRNA ENST869 which affects the Nestin promoter activity.

**Table 2:** The primers used for subcloned plasmids are summarized.

Gene Name	Primer Orientation	Sequence
Nestin P1	Forward	5’-CCCTAACCTTCCCTCTCCTG-3’
	Reverse	5’-TGGCACTCACAGGTCTTCTG-3’
Nestin P2	Forward	5’-TCACCCAAATTTTCCCTCAC-3’
	Reverse	5’-CTTAGGCACTCAGGGCACTC-3’
Nestin P3	Forward	5’-AGGAGGCAGAGATGCAAGAA-3’
	Reverse	5’-TGTGGGAGGTCACACTGGTA-3’
Nestin P4	Forward	5’-CTGGGGCCAGAGTAGATCCT-3’
	Reverse	5’-AGGCAGAGGCAGACACAGAT-3’
Nestin P4-1	Forward	5’-CTGGGGCCAGAGTAGATCCT-3’
	Reverse	5’-TAAACTCCACCTCAGGGAAC-3’
Nestin P4-2	Forward	5’-GATAGTATATTGGATTCCCC-3’
	Reverse	5’-GAAACCCAACAAAGCCAGGA-3’
Nestin P4-3	Forward	5’-CTGGGGAAGGCGGAGCTGGT-3’
	Reverse	5’-AGGCAGAGGCAGACACAGAT-3’
	Reverse	5’-ACGACCAAATCCGTTGACTC-3’

### LncRNA ENST869 Does Not Directly Bind to Nestin Protein

**Figure 6:**
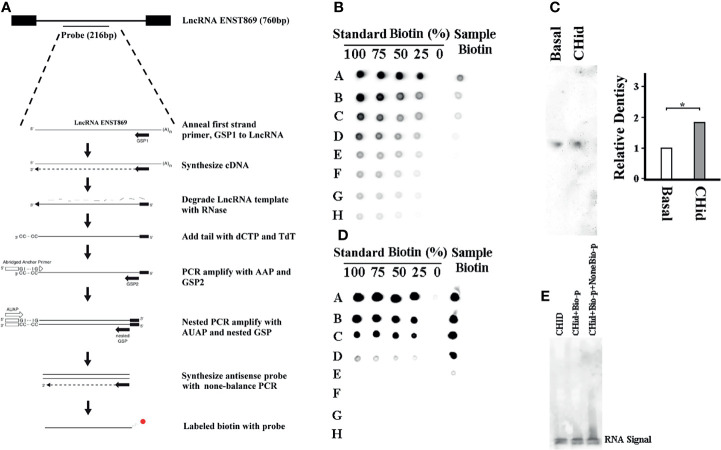
LncRNA ENST869 does not directly bind to Nestin protein. 5’RACE was used for rapid amplification of antisense LncRNA ENST869 probe and labeled with biotin **(A)**. Dot blot hybridization was used to determine the probe activity **(B)** and Northern blot was used to detect the expression level of LncRNA ENST869 in the MDA-MB-231 cells treated with Chidamide **(C)**. The direct interaction between LncRNA and Nestin protein, RNA-Protein pulldown and EMSA assay **(D**, **E)**. Data was presented as means ± S.D. for three independent experiments and analyzed using SPSS software, *20 μM Chidamide versus Basal at *p <*0.05.

Finally, to find the direct evidence of the interaction between LncRNA ENST869 and Nestin, we observed the induction effect of Chidamide on LncRNA ENST869 accumulation at the RNA level. As shown in **Figure 6A**, we labeled biotin with an antisense ENST869 probe synthesized by 5’RACE and then determined the probe hybridized activity by dot blot (**Figure 6B**). Then, the probe with 100% activity was hybridized with the RNA extracted from cells on a positive nylon membrane. As shown with the Northern blot in **Figure 6C**, the expression level of LncRNA ENST869 with Chidamide treatment was notably higher than that in the control group. While we synthesized the full sequence of LncRNA ENST869 as a probe to incubate with the total protein extracted from Chidamide treated cells. RNA-Protein Pulldown and EMSA assay showed no evidence of LncRNA ENST869 binding with any protein directly (**Figures 6D, E**). Western blot data also could not detect Nestin protein in the flow-through (data not shown).

## Discussion

Breast cancer is a malignant tumor occurring in the epithelia of the mammary gland (17, 18), Its occurrence and development are not only related to hereditary and endocrine factors, but also related to abnormal gene expression (19). HDACi is a family of anti-cancer drugs with great clinical application prospects. It mainly affects the corresponding target gene expression by changing the spatial structure of histone, and plays an important role in regulating cancer cell growth and cell cycle progression (20–24). Chidamide, as a new generation HDACi, has shown exceptional anti-breast cancer effects in previous experiments (25–28). In this paper, the treatment of Chidamide on MCF-7 or MDA-MB-231 breast cancer cells also confirmed that it could reduce the vitality of breast cancer cells and inhibit the growth of cancer cells.

With the increasing research on gene functions, LncRNA has attracted more attention. Its unique approach in influencing gene expression has been realized (29–32). Here, we screened the expression of LncRNA ENST869 by high-throughput sequencing combined with bioinformatics analysis and real-time PCR verification. The data showed that LncRNA ENST869 induced by Chidamide was relevant to Nestin function. Nestin is a type VI intermediate filament protein (33). There is clear evidence showing that the expression of Nestin is increased in a variety of tumor tissues including breast cancer, and is closely related to tumor malignancy. Therefore, it can be used as a biomarker to predict cancer occurrence and development (34).

Anti-cell death is an important feature of tumor cell survival (35). It enables tumor cells to withstand various challenges, including chemotherapeutic drugs (36). Nestin plays an essential role in the resistance against conventional chemotherapy of tumor stem cells (37–39). Nestin prevents tumor cell death by inducing DNA damage repair (40, 41). Nestin positive cells mark the existence of a stem cell-like population, which enables tumor cells to continue to survive and differentiate (42–44). For example, in glioma, Nestin positive cells labeled with stem cell like populations enable tumor cells to survive and proliferate when exposed to chemotherapeutic drugs (45, 46). Silencing Nestin expression with shRNA in nasopharyngeal carcinoma cells interrupted doxorubicin-induced DNA damage repair (47, 48). Studies also have shown that Nestin knockout by some LncRNAs increased cells sensitive to X-ray radiation in xenotransplantation experiments (49). In our experiments, the anti-breast cancer drug Chidamide acts on breast cancer cells to activate Nestin expression through LncRNA ENST869, and this “inappropriate expression” is just the result of self-protection of cancer cells against drugs, suggesting that Nestin may participate in the pharmacological action against Chidamide, which may be the key in breast cancer cell drug resistance.

Bioinformatic analysis indicated that there is an interaction between LncRNA ENST869 and Nestin. We evaluated this interaction through RNA protein pull-down and other experiments. The results showed that the expression level of ENST869 RNA after Chidamide treatment was higher than that in the control group. LncRNA ENST869 did not bind directly to any protein. It is well known that LncRNA can participate in the regulation of target genes at different levels, namely, pre-transcription, transcription, and post-transcription (50–54). Luciferase assay showed that LncRNA ENST869 might regulate Nestin mRNA transcription through classical anchorage of the transcriptionally active region. Because the common mode of most LncRNAs is to influence the transcription of downstream genes, it means that the direct regulation of Nestin transcription by LncRNA ENST869 might form as an integration platform, polymerizing transcription elements including transcription factors around LncRNAs to realize the transmission of upstream and downstream effector molecules. This can purposefully locate the transcription complex on the specific sequence of the promoter in an accurate pattern. Notably, studies have shown that other LncRNAs such as MALAT1 and Rik-203 may also play roles in the network affecting Nestin (55, 56).

**Figure 7:**
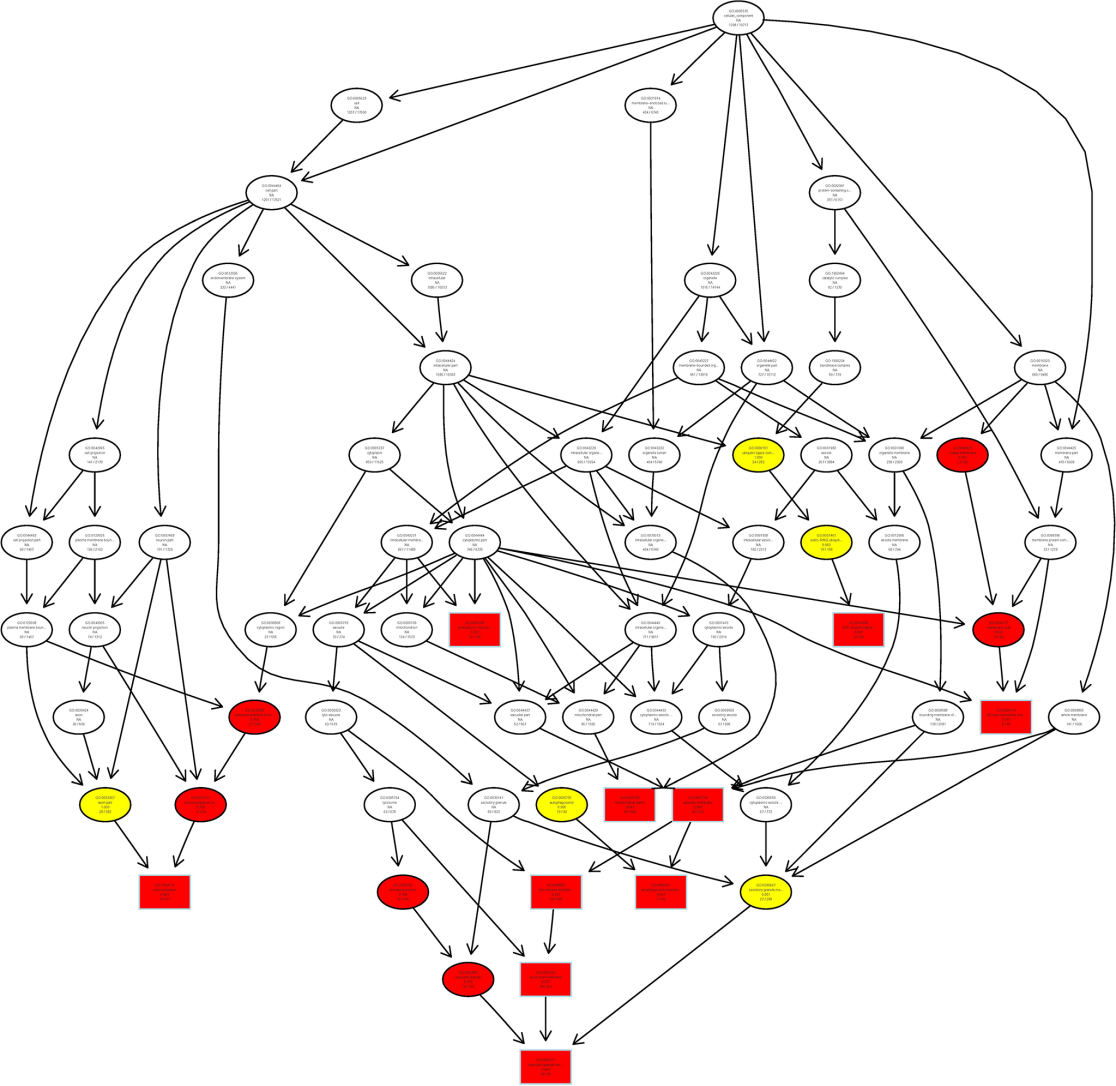
Schematic representation of co-expression network of differentially expressed LncRNAs/mRNAs. All differentially expressed LncRNAs/mRNAs were selected for GO pathway analyses. GO was performed with KOBAS 3.0 software. GO analysis covered the cellular component (CC) domains.

Finally, we analyzed the target gene by the Gene Ontology (GO) enrichment function of LncRNA from Chidamide treated MDA-MB-231 breast cancer cells, and found that most LncRNAs in cells treated with Chidamide were mostly involved in autophagy initiation and the formation of secondary autophagic bodies (**Figure 7**), which is consistent with our previous experimental findings (16). That is, Chidamide exerts its pharmacological effect mainly by stimulating excessive autophagic cell death.

## Conclusion

Our study demonstrated that the induction of LncRNA ENST869 induced the death of breast cancer cells by regulating Nestin expression that was activated by Chidamide treatment. During this process, Nestin is involved in the pharmacological action of Chidamide against breast cancer cells. Nestin is the main factor in driving drug resistance. More detailed and specific mechanism between LncRNA ENST869 and Nestin needs further experimental verification.

## Data Availability Statement

The datasets presented in this study can be found in online repositories. The names of the repository/repositories and accession number(s) can be found below: the raw RNA-seq data is available in NCBI under the Sequence Read Archive (SRA) with accession number PRJNA807845. The data related to RNA-seq results were displayed in **Supplementary Materials**.

## Ethics Statement

The animal study was reviewed and approved by the Shenyang Medical College.

## Author Contributions

WZ and XyF participated in the design of the study. WZ, HH, YG, and XyF performed the experiments. WZ, HH, and XyF analyzed data. WZ wrote the manuscript. All authors listed have made a substantial, direct, and intellectual contribution to the work and approved it for publication.

## Funding

The work is supported by the Natural Science Foundation of China (81172509), the Research Foundation of Education Department of Liaoning Province (SYYX202004, 2021010620-301), the Natural Science combination Foundation for improving innovation of Liaoning Province (2022012046-301), and the Research Foundation of Shenyang Science and Technology Bureau (20-205-4-066).

## Conflict of Interest

The authors declare that the research was conducted in the absence of any commercial or financial relationships that could be construed as a potential conflict of interest.

## Publisher’s Note

All claims expressed in this article are solely those of the authors and do not necessarily represent those of their affiliated organizations, or those of the publisher, the editors and the reviewers. Any product that may be evaluated in this article, or claim that may be made by its manufacturer, is not guaranteed or endorsed by the publisher.
