# Supplementary material for: LncRNA ENST869 Targeting Nestin Transcriptional Region to Affect the Pharmacological Effects of Chidamide in Breast Cancer Cells
Source: Front Oncol. 2022 Apr 4;12:874343. doi: 10.3389/fonc.2022.874343 (PMC9014306; doi:10.3389/fonc.2022.874343)
Supplement: Supplementary file 1 [file DataSheet_1.pdf]

Nestin Promoter Fragment Cloning for Luciferase Assay

1. Fragment PCR Amplification from Genomic DNA

(Primers containing PmeI and NotI enzyme site)

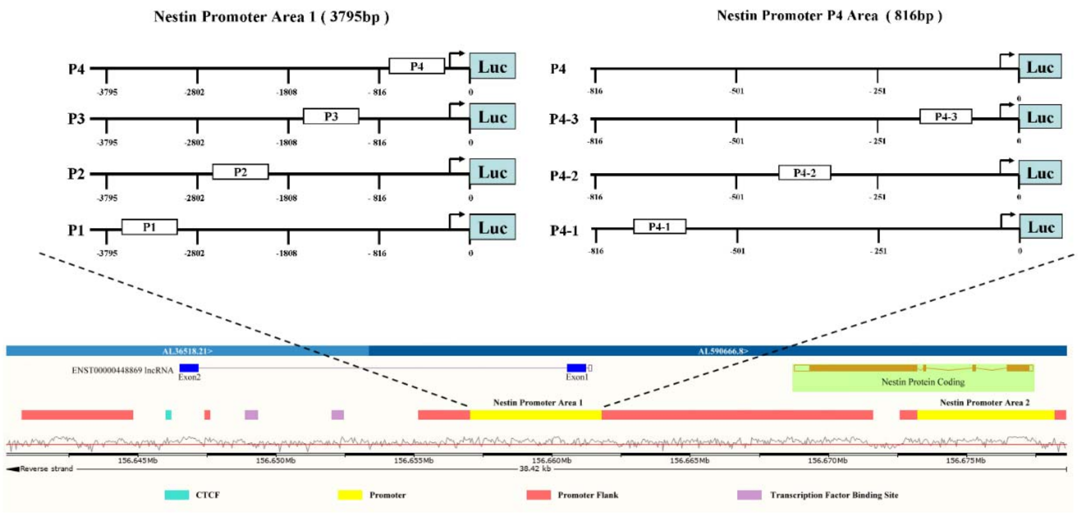

P1 P2 P3

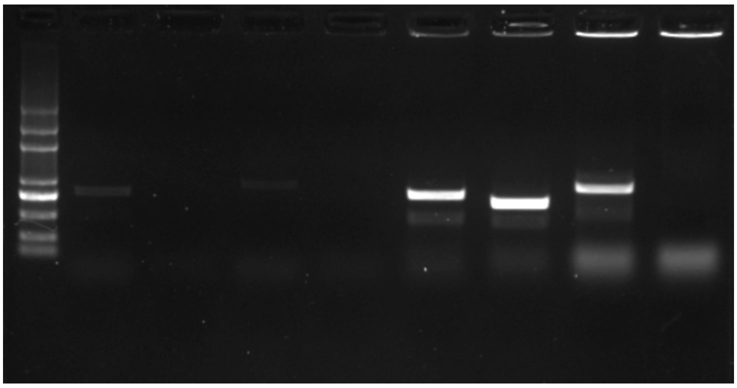

P4

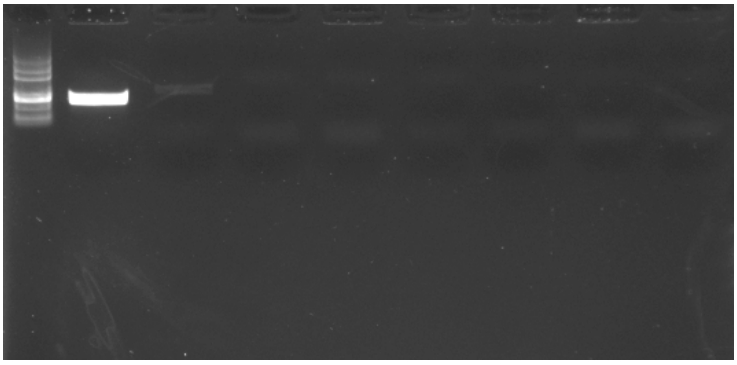

P4-1 P4-2 P4-3

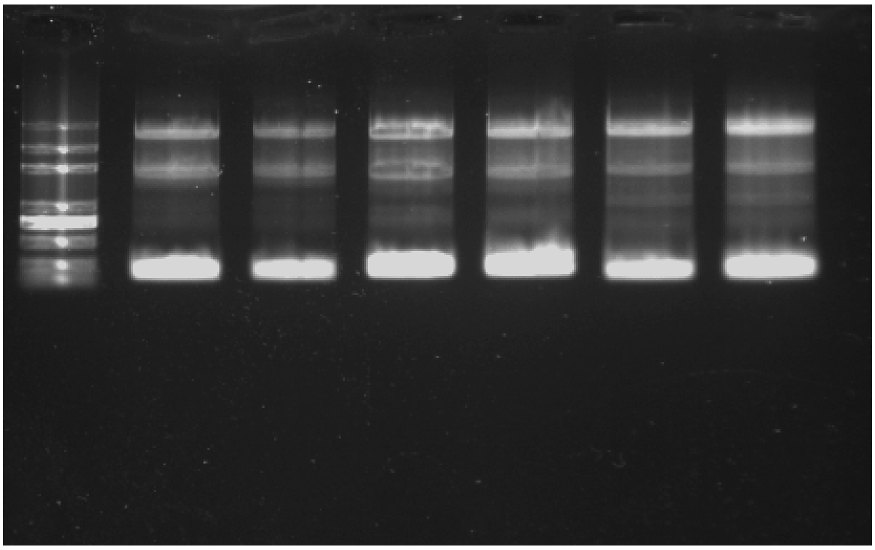

←

2. Cloning PCR for Screening sense colony.

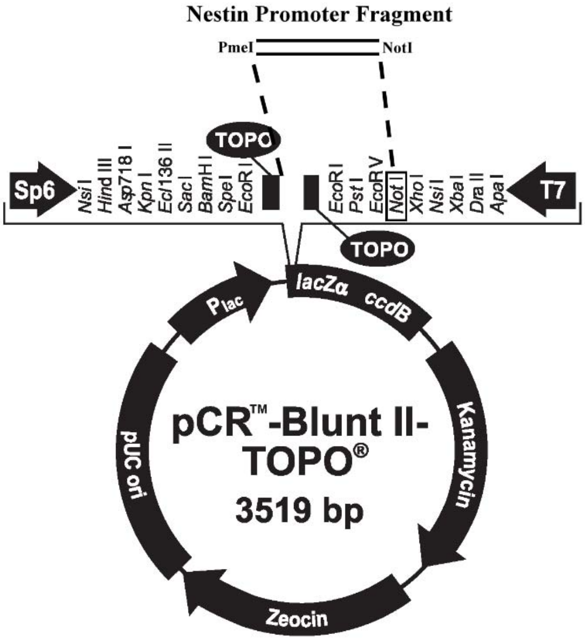

P4-1-TOPO p4-2-TOPO p4-3-TOPO

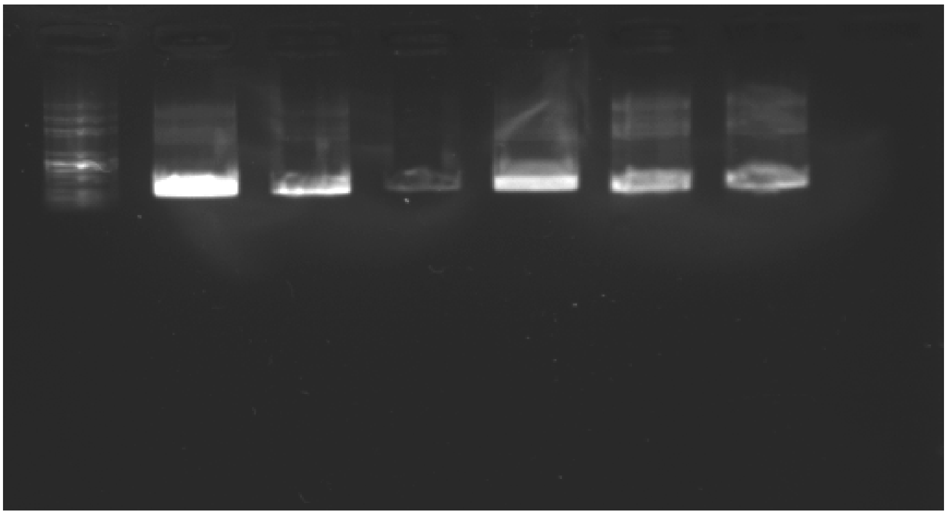

P1-TOPO P2-TOPO P3-TOPO P4-TOPO

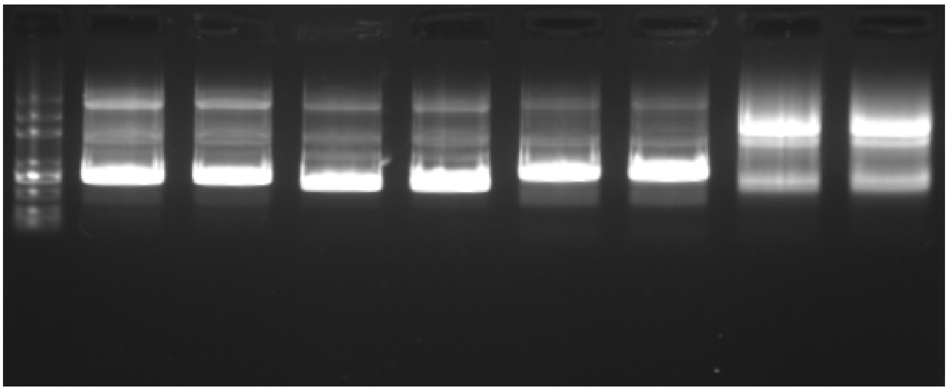

3. PmiI and NotI enzyme cite the plasmids

P1 P2 P3 P4 P4-1 P4-2 P4-3 psiCHECK

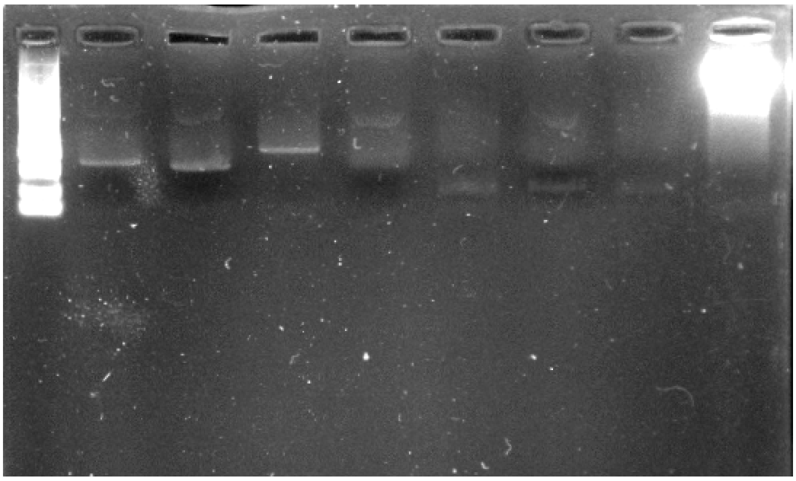

4. **Ligation Nestin promoter fragments with psiCHECK fragment, after transformation, make colony PCR to verify the positive plasmid.**

P1

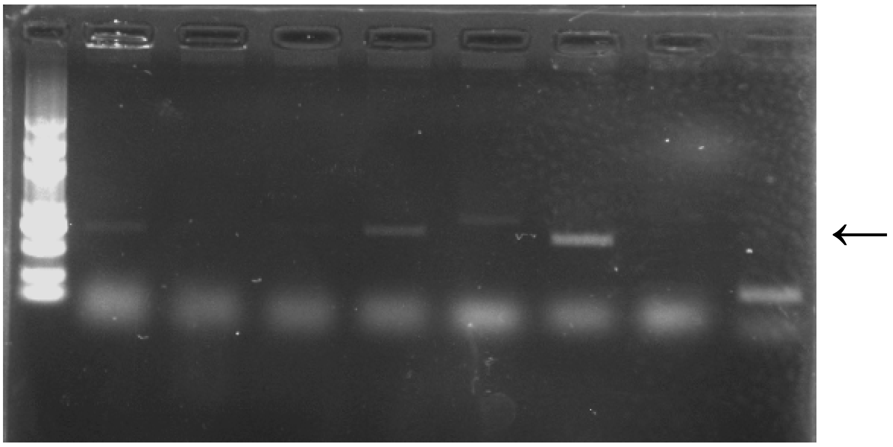

P2

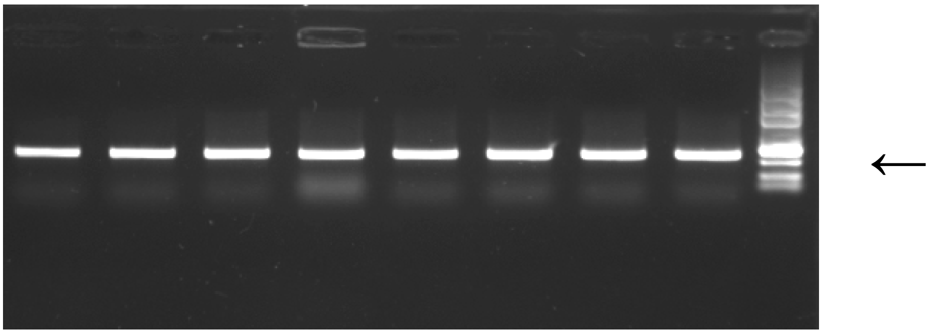

P3

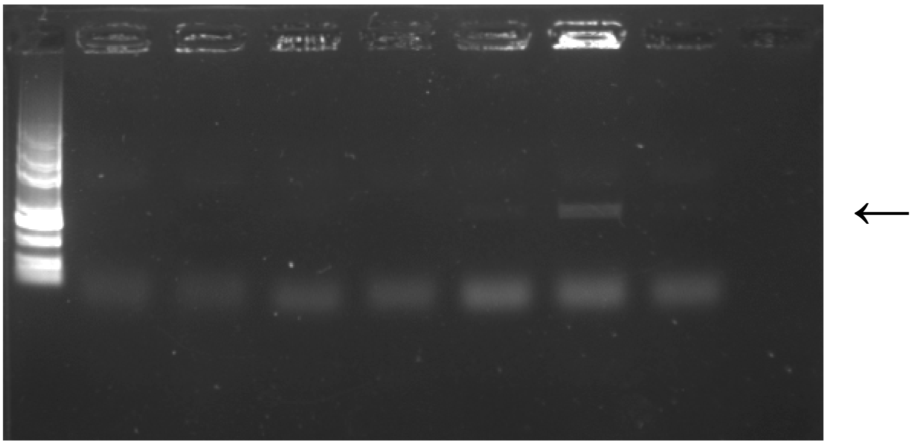

P4

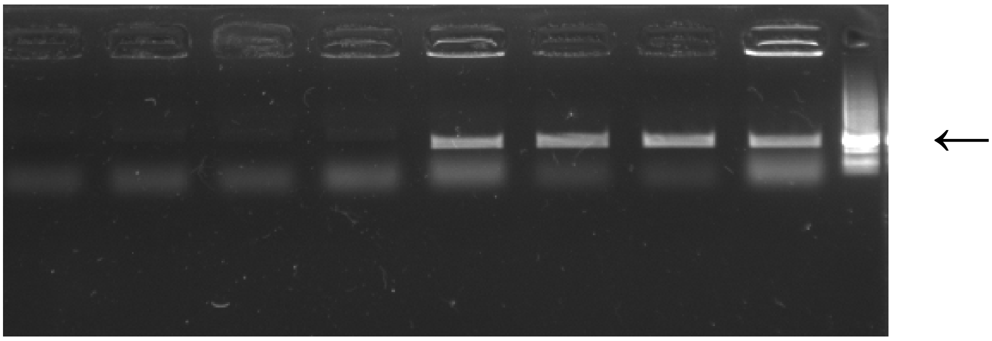

P4-1

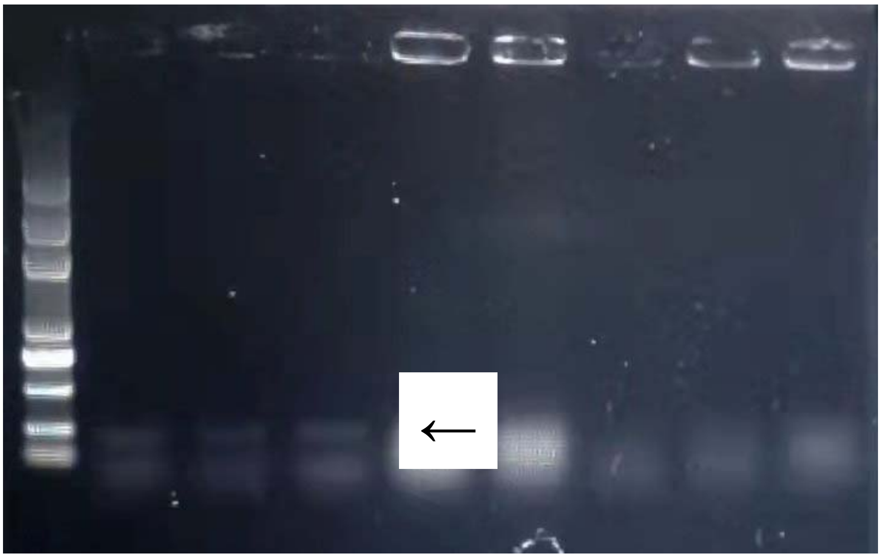

P4-2

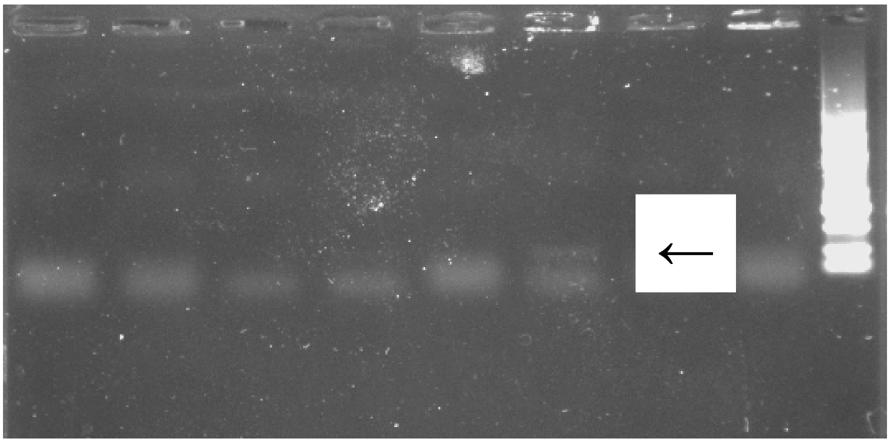

P4-3

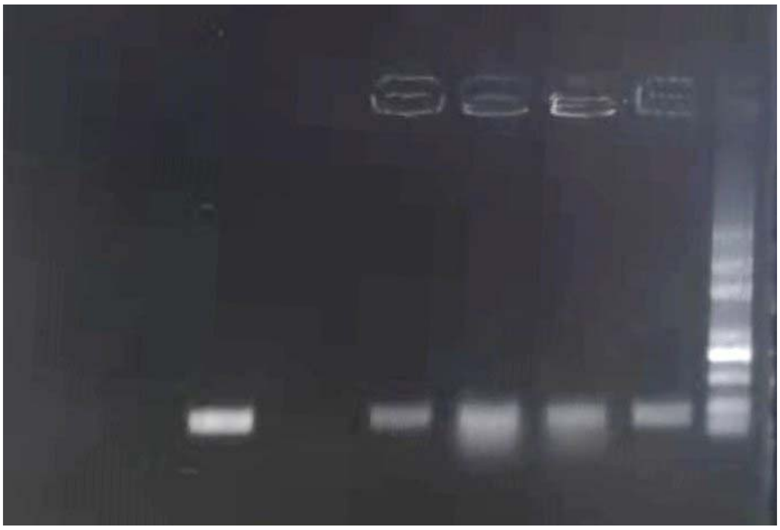

←
