# Supplementary material for: LncRNA ENST869 Targeting Nestin Transcriptional Region to Affect the Pharmacological Effects of Chidamide in Breast Cancer Cells
Source: Front Oncol. 2022 Apr 4;12:874343. doi: 10.3389/fonc.2022.874343 (PMC9014306; doi:10.3389/fonc.2022.874343)

Figure3

MCF-7 Actin

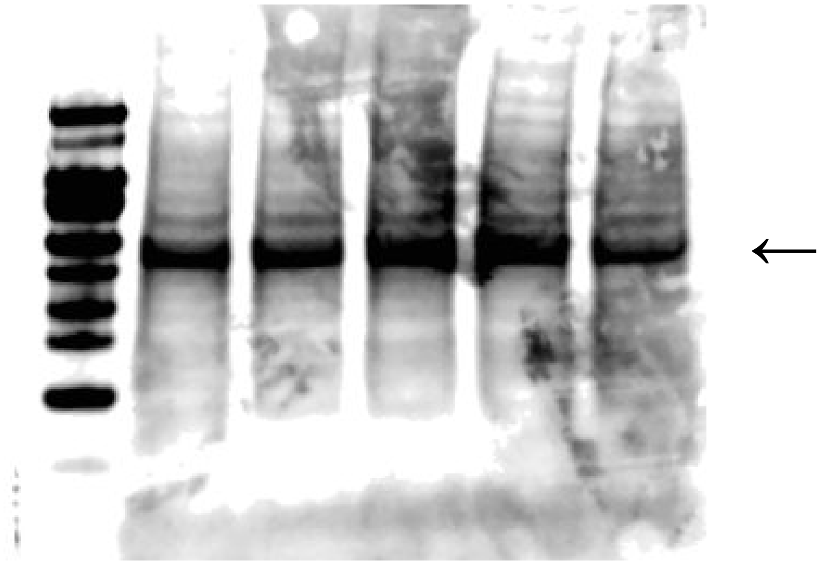

MCF-7 Nestin

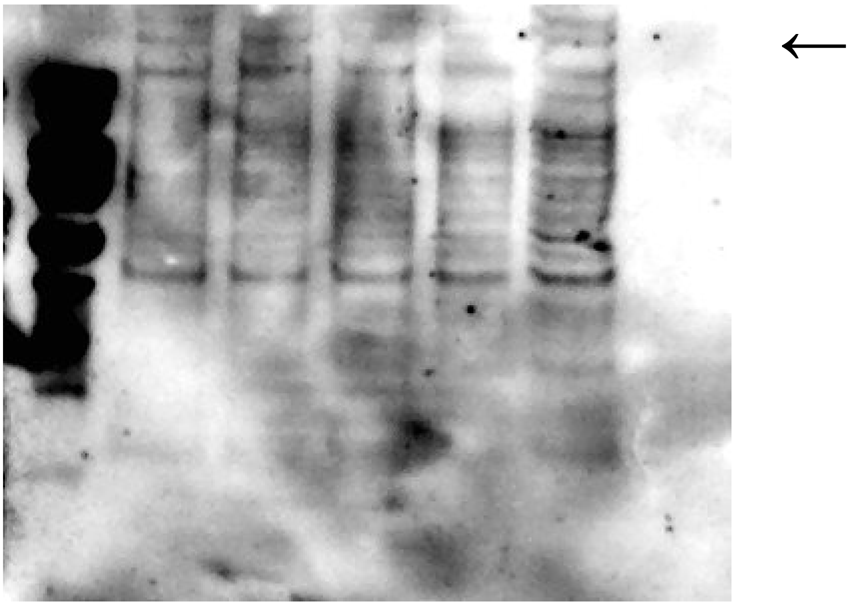

MDA-MB-231 Actin

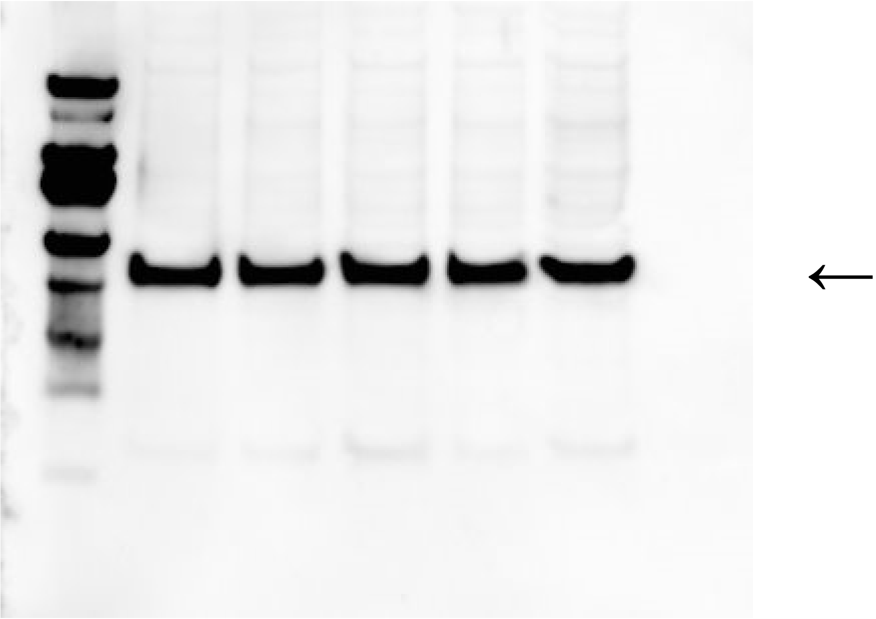

MDA-MB-231 Nestin

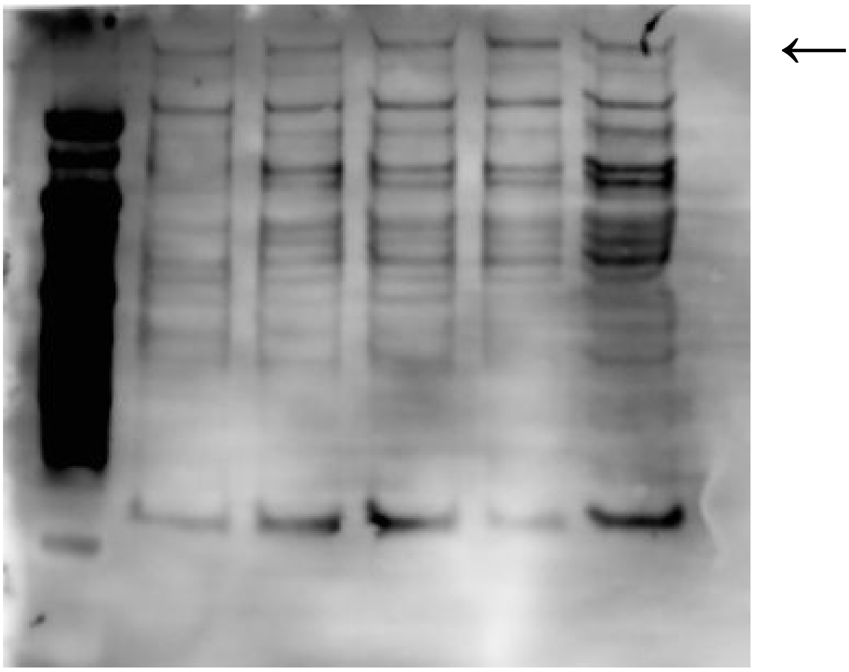

Figure 4

MCF-7 Actin

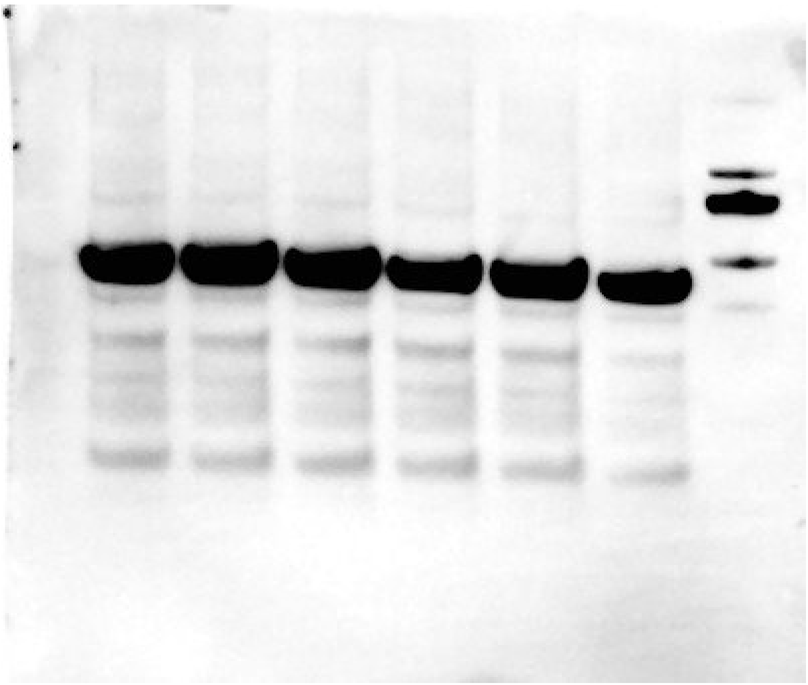

←

MCF-7 Nestin

←

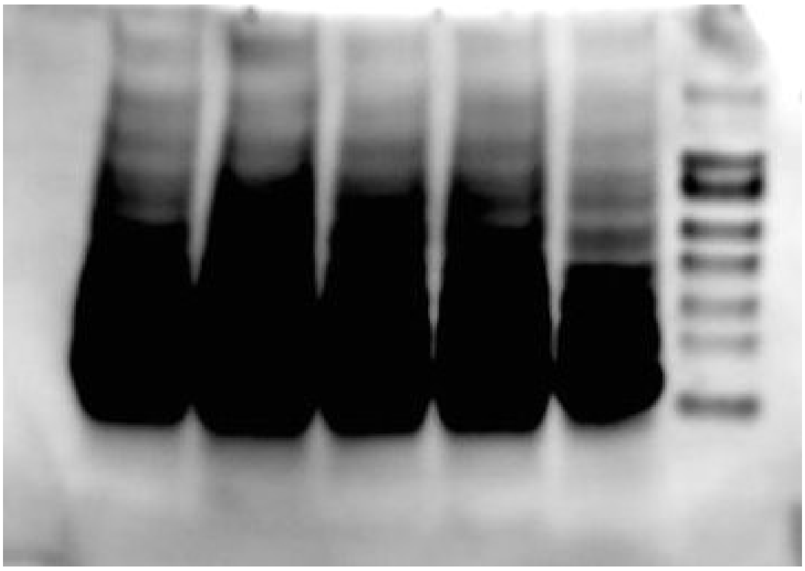

MDA-MB-231 Actin

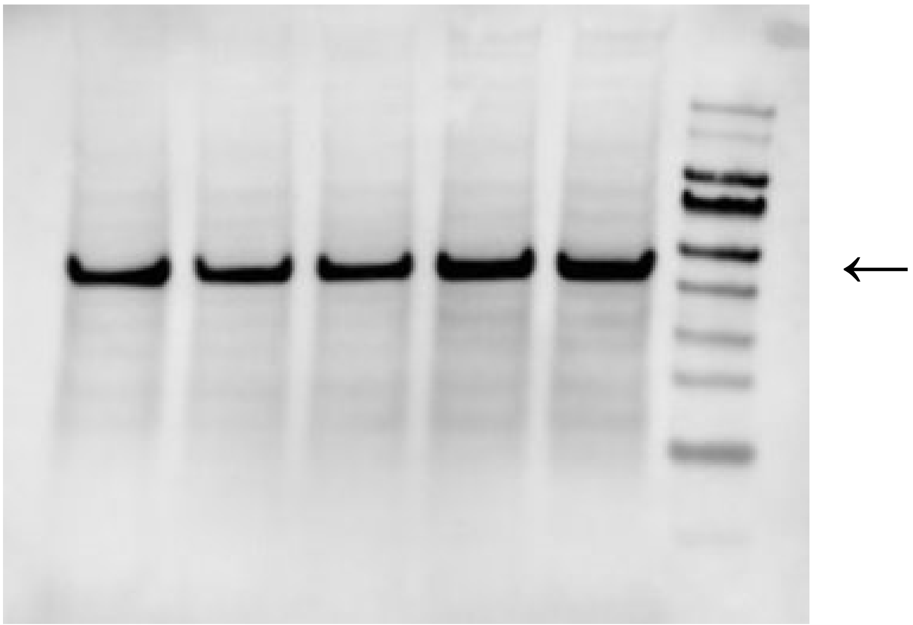

MDA-MB-231 Nestin

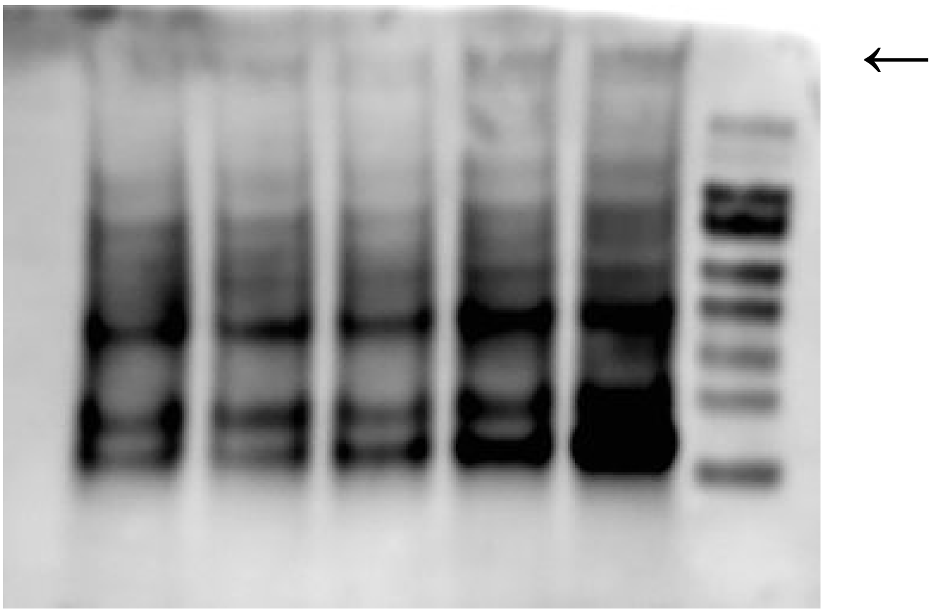

Supplement: Supplementary file 2 [file DataSheet_2.pdf]
