# Supplementary material for: LncRNA ENST869 Targeting Nestin Transcriptional Region to Affect the Pharmacological Effects of Chidamide in Breast Cancer Cells
Source: Front Oncol. 2022 Apr 4;12:874343. doi: 10.3389/fonc.2022.874343 (PMC9014306; doi:10.3389/fonc.2022.874343)

MCF7 [MCF-7] cells STR report

Method: An appropriate amount of MCF7 [MCF-7] cells (Cell number PC75,1×106) was used to extract DNA, 20 STR loci and gender identification loci were amplified by 21 CELLID System, PCR product detection was performed by ABI3130x1 genetic analyzer, detection results were analyzed by GeneMapper IDX software (Applied Biosystems), and compared with ATCC, DSMZ, JCRB, Cellosaurus and other databases.

Experimental result:

1. The results of negative and positive control were correct.
2. The genotyping results of STR locus of MCF7 [MCF-7] cell line are shown in the following table.

Conclusion:

1. The genomic DNA of MCF7 [MCF-7] cell line is clear and the result of genotyping is good.
2. The results of STR typing showed that no cross contamination of mouse cells and human cells was found in the cell line of MCF7 [MCF-7] cell line.
3. The DNA typing of the cell line was 96.88% matched with the cell type in the cell bank, and the cell line name was MCF7 [MCF-7].

Appendix I: The genotyping results of STR locus of MCF7 [MCF-7] cell line

STR Loci	Sample：PC75	Database：MCF7 [MCF-7]
Amelogenin	X	X
CSF1PO	10	10
D2S1338	21,23	21,23
D3S1358	16	16
D5S818	11,12	11,12
D7S820	8,9	8,9
D8S1179	10,14	10,14
D13S317	11	11
D16S539	11,12	11,12
D18S51	14	14
D19S433	13	13,14
D21S11	30	30
FGA	23,25	23,25
PentaD	12	12
PentaE	7,12	7,12
TH01	6	6
TPOX	9,12	9,12
vWA	14,15	14,15
D1S1656	11,15.3	
D6S1043	12,18	
D12S391	18,20	
D2S411		
The Cellosaurus database has a matching rate of 96.88%，The number of matched bits is 20（https://web.expasy.org/cellosaurus-str-search/）		

Note:

1. According to the cell STR identification standard established by the International Cell Line Authentication Committee (ICLAC), when the matching degree of cell lines is ≥80%, they are considered to be correlated, that is, derived from common ancestral cells; The matching degree is between 55% and 80%, and the correlation needs to be further verified. Less than 55% indicates no correlation between the two.
2. The effective peak of the map was the real PCR band; Small peaks and nonspecific bands were ignored in the calculation.

Appendix III: The genotyping results of STR locus of MCF7 [MCF-7] cell line

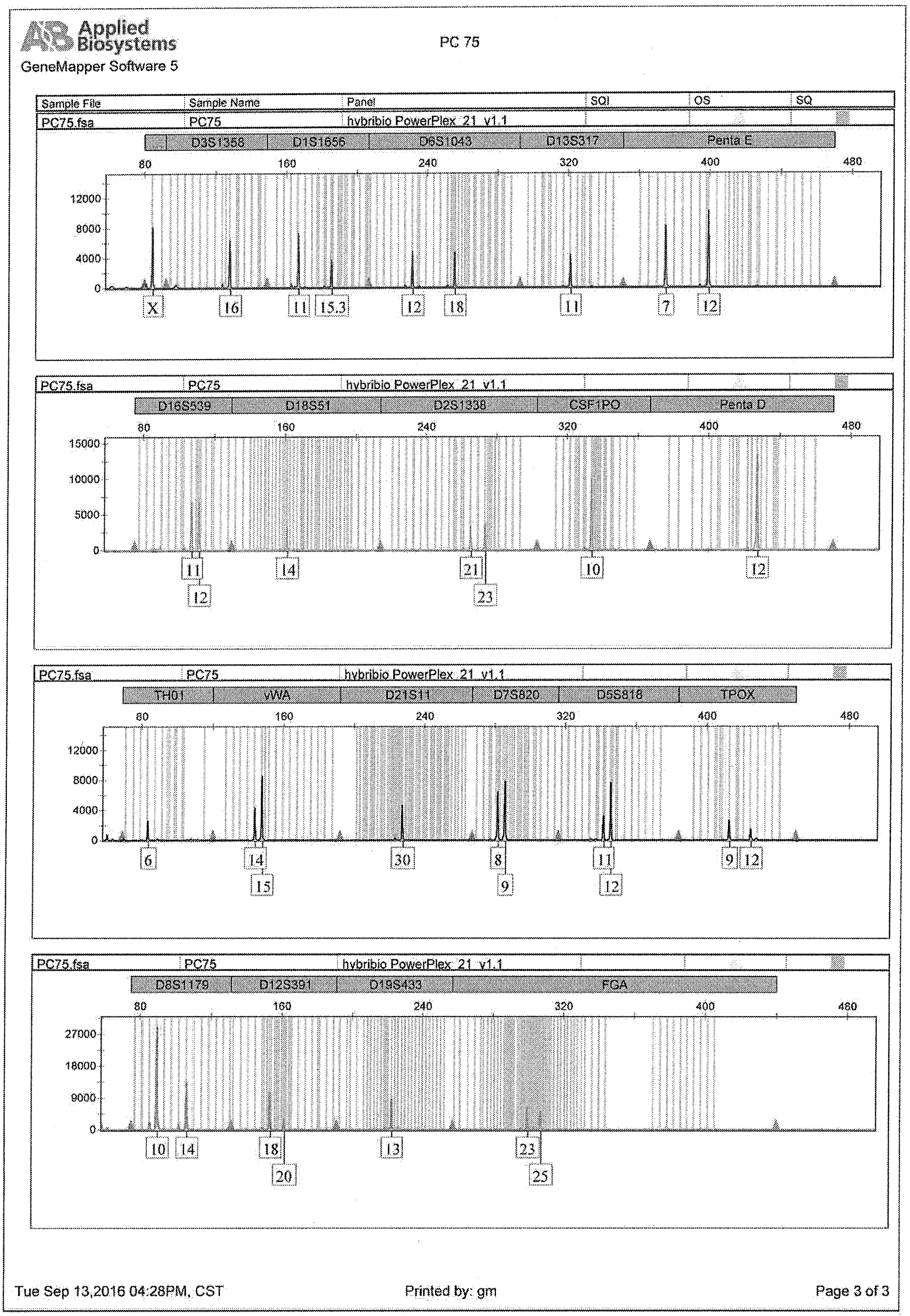

Supplement: Supplementary file 3 [file DataSheet_3.pdf]
