# Supplementary material for: LncRNA ENST869 Targeting Nestin Transcriptional Region to Affect the Pharmacological Effects of Chidamide in Breast Cancer Cells
Source: Front Oncol. 2022 Apr 4;12:874343. doi: 10.3389/fonc.2022.874343 (PMC9014306; doi:10.3389/fonc.2022.874343)

MDA-MB-231cells STR report

Method: An appropriate amount of MDA-MB-231 cells (Cell number PC03,1×106) was used to extract DNA, 20 STR loci and gender identification loci were amplified by 21 CELLID System, PCR product detection was performed by ABI3130x1 genetic analyzer, detection results were analyzed by GeneMapper IDX software (Applied Biosystems), and compared with ATCC, DSMZ, JCRB, Cellosaurus and other databases.

Experimental result:

1. The results of negative and positive control were correct.
2. The genotyping results of STR locus of MDA-MB-231cell line are shown in the following table.

Conclusion:

1. The genomic DNA ofMDA-MB-231 cell line is clear and the result of genotyping is good.
2. The results of STR typing showed that no cross contamination of mouse cells and human cells was found in the cell line of MDA-MB-231cell line.
3. The DNA typing of the cell line was 100.00% matched with the cell type in the cell bank, and the cell line name wasMDA-MB-231.

Appendix I: The genotyping results of STR locus ofMDA-MB-231 cell line

STR Loci	Sample：PC03	Database：MDA-MB-231
Amelogenin	X	X
CSF1PO	12,13	12,13
D2S1338		20,21
D3S1358	16	16
D5S818	12	12
D7S820	8,9	8,9
D8S1179	13	13
D13S317	13	13
D16S539	12	12
D18S51	11,16	11,16
D19S433		11,14
D21S11	33.2	33.2
FGA	22,23	22,23
PentaD	11,14	11,14
PentaE	11	11
TH01	7,9.3	7,9.3
TPOX	8,9	8,9
vWA	15,18	15,18
D1S1656		
D6S1043		
D12S391		
D2S411		
The Cellosaurus database has a matching rate of 100.00%，The number of matched bits is 15 （https://web.expasy.org/cellosaurus-str-search/）		

Note:

1. According to the cell STR identification standard established by the International Cell Line Authentication Committee (ICLAC), when the matching degree of cell lines is ≥80%, they are considered to be correlated, that is, derived from common ancestral cells; The matching degree is between 55% and 80%, and the correlation needs to be further verified. Less than 55% indicates no correlation between the two.
2. The effective peak of the map was the real PCR band; Small peaks and nonspecific bands were ignored in the calculation.

Appendix III: The genotyping results of STR locus of MDA-MB-231 cell line

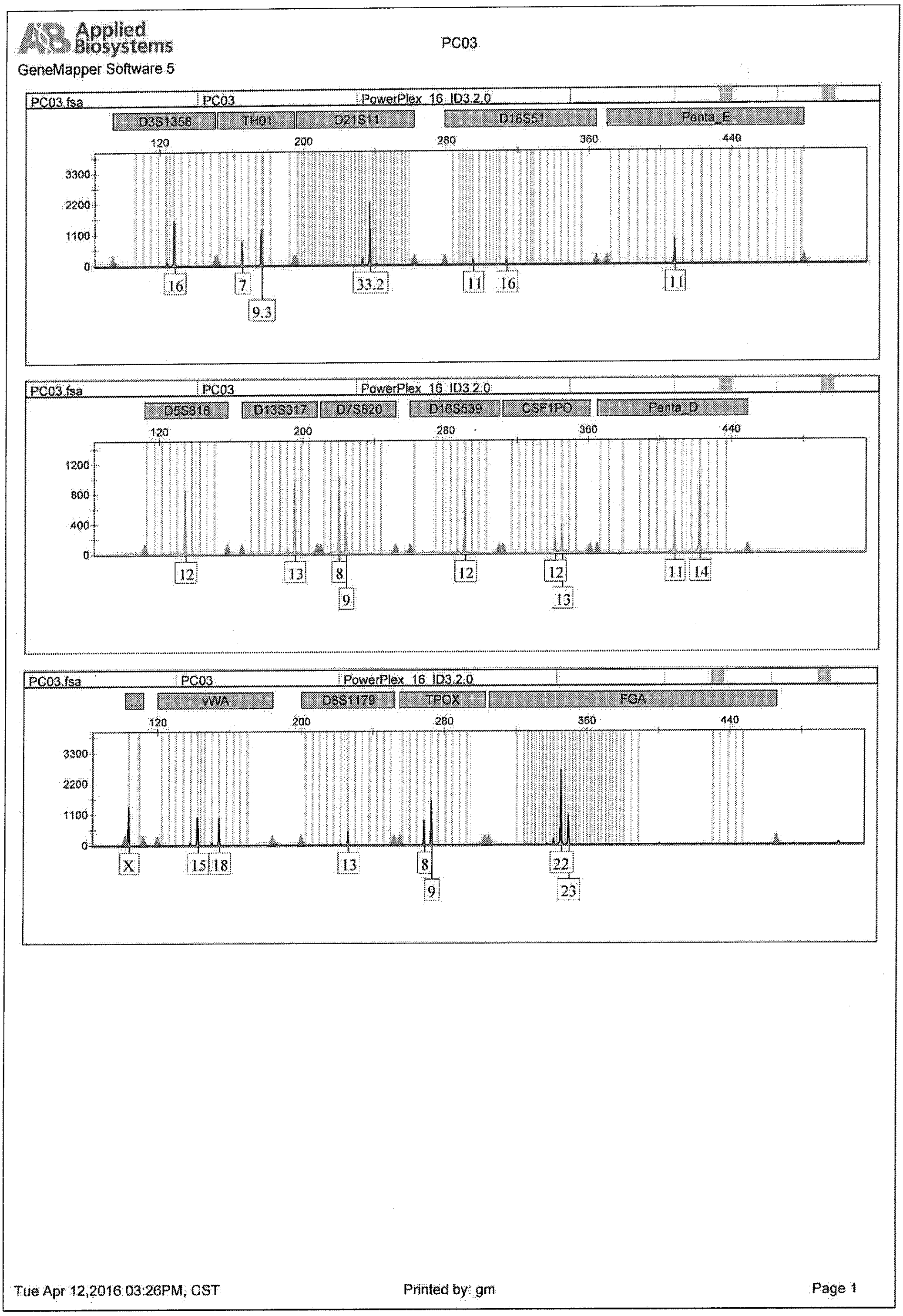

Supplement: Supplementary file 4 [file DataSheet_4.pdf]
